# Supplementary material for: Nanoconfinement-triggered oligomerization pathway for efficient removal of phenolic pollutants via a Fenton-like reaction
Source: Nat Commun. 2024 Jan 31;15:917. doi: 10.1038/s41467-024-45106-4 (PMC10831074; doi:10.1038/s41467-024-45106-4)
Supplement: Supplementary file 1 — Supplementary Information [file 41467_2024_45106_MOESM1_ESM.pdf]

Supplementary information for

Nanoconfinement-triggered oligomerization pathway for the efficient removal of phenolic pollutants via a Fenton-like reaction

Xiang Zhang[1,5], Jingjing Tang[1,5], Lingling Wang[1,5], Chuan Wang[1], Lei Chen[2], Xinqing Chen[4], Jieshu Qian[1,2,3*], and Bingcai Pan[2*]

[1] Jiangsu Key Laboratory of Chemical Pollution Control and Resources Reuse, School of Environmental and Biological Engineering, Nanjing University of Science and Technology, Nanjing 210094, China

[2] Research Center for Environmental Nanotechnology (ReCENT), School of Environment, Nanjing University, Nanjing 210023, China

[3] School of Environmental Engineering, Wuxi University, Jiangsu 214105, PR China.

[4] CAS key Laboratory of Low-carbon Conversion Science and Engineering, Shanghai Advanced Research Institute, Chinese Academy of Sciences, Shanghai 201210, China

[5] These authors contributed equally: Xiang Zhang, Jingjing Tang, and Lingling Wang.

*Corresponding authors: Jieshu Qian (qianjieshu@foxmail.com) and Bingcai Pan (bcpan@nju.edu.cn)

This PDF file includes:

Supplementary methods

Supplementary Note 1

Supplementary Figure 1 to 36

Supplementary Table 1 to 5

Supplementary methods

The concentration of Fe ions was examined by ICP-MS (NexION 300D, PerkinElmer).

XRD patterns were obtained using X′Pert PRO, PANalytical, Netherlands, using monochromatic Cu K_{α} radiation (λ = 0.154 nm) in a 2θ scan range of 5°-30°.

The TEM, HRTEM, SAED, and EDS mapping images were obtained using a Tecnai G2 F30 S-Twin, FEI, operating at voltage of 200 kV.

Field emission SEM images were acquired by JSM-IT500HR, Japan.

Raman spectra were acquired by an Aramis type confocal laser micro-Raman spectrometer (HORIBA JOBIN YVON, France) equipped with a He-Ne laser of excitation of 532 nm.

XPS was performed on ULVAC-PHI X-TOOL, Japan, using the Al K_{α} line (1486.7 eV) as an excitation source.

The Mössbauer spectra were measured by a MFD-500AV-01 type in-situ/operado ^{57}Fe Mössbauer spectrometer (Topologic Systems, Japan).

The EXAFS spectra were recorded at Shanghai Synchrotron Radiation Facility (SSRF, China). X-rays from an undulator were monochromatized by a Si (111) double-crystal monochromator. The acquired EXAFS data were processed according to the standard procedures using the ATHENA module implemented in the IFEFFIT software packages. The k^3-weighted EXAFS spectra were obtained by subtracting the post-edge background from the overall absorption and then normalizing with respect to the edge-jump step. Subsequently, k^3-weighted $\chi(k)$ data of were Fourier transformed to real (R) space using a hanning windows (dk = 1.0 Å^{-1}) to separate the EXAFS contributions from different coordination shells. To obtain the quantitative structural parameters around central atoms, least-squares curve parameter fitting was performed using the ARTEMIS module of IFEFFIT software packages (1, 2, 3).

The concentrations of pollutant were determined using a HPLC (Thermo Fisher Scientific UltiMate 3000 Series) system equipped with a C-18 column (4.6 mm×150 mm×5 μm) with the column temperature at 30 °C and a UV detector (G1314F 1260VWD). The mobile phase was a mixture of 0.1% (v/v) aqueous formic acid solution and MeOH (50/50, v/v) for phenol and 4-CP, (30/70, v/v) for HQ, PBQ. The mobile phase was a mixture of water and methanol (35/65, v/v) for BPA. The mobile phase was a mixture of water and acetonitrile (75/25, v/v) for PMSO and $PMSO_2$.

Reactive species of •OH and $^1O_2$ in solution were detected using DMPO and

TEMP, respectively, as spin-trapping reagents using an electron paramagnetic spectrometer (EPR, Bruker A300, Bremen, Germany).

The contribution of $O_2^{\bullet-}$ using NBT as the chemical probe was evaluated by the ultraviolet visible spectrophotometer (UV-vis, UV-2600i, Shimadzu, Japan).

The degradation intermediates of phenol were detected by UHPLC (U3000, Thermo) coupled with a high resolution Q Exactive Focus Orbitrap tandem mass spectrometer (Thermo Fisher scientific Inc., Germany) with C-18 column (2.7 μm, 2.1 × 100mm). (A) Water/(B) MeOH was used as mobile phase at a flow rate of 0.2 mL min^{-1}. The column temperature was set at 35 ºC. Mobile phase procedure followed a gradient elution: 2% B for in the first 2 min, then linearly increase from 2 % B to 50 % B in 10 min, then maintained at 50 % B for 6 min, and then reduced to 2 % B in 0.5 min, at last kept at 2 % B for 2.5 min. Mass spectral analysis was conducted in positive mode over a mass range of 50~500 m/z.

The ring-opening products were analyzed by GC-MS (TRACE1310/ISQ, Thermo Scientific, United States) with a TG-5 MS column (30 m × 0.25 mm × 0.25 μm, Thermo Scientific, United States). Prior to injection, the samples were quenched by MeOH and evaporated using a freeze-drying method. Then, 2 mL of dichloromethane were added to the residues. After being dehydrated by anhydrous $MgSO_4$, the sample derivatization was carried out using HMDS (0.10 mL) and TMSCl (0.05 mL) for 5 min at room temperature. The precipitates were separated by centrifugation before analysis. Chromatographic parameters are as follows: the injector temperature of 250 °C; an initial oven temperature of 40 °C for 2 min, then programmed increased to 300 °C at a rate of 10 °C/min and held for 2 min.

The oligomerized products were analyzed using MALDI-TOF MS (TOF-4800 plus, ABSCIEX) using 2,5-dihydroxybenzoic acid (DHB) in positive mode over a mass range of 250~750 m/z.

Chronoamperometry curves were recorded on a three-electrode electrochemical workstation (CHI 730E, Shanghai Chenhua Inc., China) equipped with an Ag/AgCl electrode as reference electrode, a Pt electrode as counter electrode, and a glassy carbon electrode (GCE) as working electrode, respectively. Chronoamperometry experiments were carried out in pH=5.0 ± 0.2 and voltage = + 0.8 V. Preparation of working electrode: 5 mg of the UiO-66-NH_2-(Zr/Fe)/GA catalyst and 20 μL of Nafion solution (5 wt. %) were dispersed in 1 mL of mixed solvent containing isopropanol and water (1:3 v/v), and a homogeneous catalyst colloid was formed by ultrasonication for 1 h.

Afterward, 15 μL of the catalyst colloid was spread on the glassy carbon electrode (GCE) and dried in air at room temperature to form a working electrode.

TOC of water samples was analyzed using TOC analyzer (Shimazu, Japan).

The concentration of H_2O_2 is measured by iodometry. Typically, 1 mL samples were withdrawn from the reactor at predetermined time intervals. The sample was filtered through a 0.22 μm polytetrafluoroethylene (PTFE) filter (ANPEL, China), followed by the addition of 1 mL 0.1 M potassium acid phthalate solution and 1 mL 0.4 M KI solution. The concentration of H_2O_2 was determined by UV-Vis spectrometer (UV-2600i, Shimadzu, Japan).

Supplementary Notes

Supplementary Note 1. Derivation of the pseudo first-order reaction kinetics model

Suppose a reaction between the reactant A and the active species B gives a product C:

$A + B \rightarrow C$ Supplementary Equation (1)

The decrease of the A concentration by B could be expressed via a second-order reaction kinetics model as:

$-d[A]/dt = k[A][B]$ Supplementary Equation (2)

where [A] is the concentration of A, [B] is the steady-state concentration of B, k is the rate constant of the reaction between A and B. It is normally assumed that [B] is irrelevant to [A] and remains constant during the reaction, so Supplementary Equation (2) is re-expressed as:

$d[A]/dt = k_{app}[A]$ Supplementary Equation (3)

$k_{app} = k[B]$ Supplementary Equation (4)

The second-order reaction kinetics model degenerates to a pseudo first-order reaction kinetics model, where k_{app} is the apparent pseudo first-order rate constant. Integrating Supplementary Equation (3) and rearranging gives:

$k_{app}= -\ln([A]/[A]_0)/t$ Supplementary Equation (5)

$[A]_0$ is the initial concentration of A. Value of k_{app} could be calculated from the slop of the plot of $-\ln([A]/[A]_0)$ vs time. All removal data in this study are fitting to Supplementary Equation (5). It is clear that the value of k_{app} should decrease as the increase of $[A]_0$, given the conditions of constant [B], representing that the steady-state concentration of [•OH] in most of the Fenton systems is constant during reaction.

However, in the conditions that the steady-state concentration of [B] is relevant to [A] and not constant, the value of [B] could be obtained from Supplementary Equation (4) and Supplementary Equation (5):

$[B] = k_{app}/k = -\ln([A]/[A]_0)/(kt)$ Supplementary Equation (6)

One could see that the value of [B] increases as the increase of k_{app}. This is the case that the steady-state concentration of [•OH] is dependent on the initial [phenol] in the UiO-66-NH_2-(Zr/Fe)/GA+H_2O_2 system and is not constant during reaction.

Supplementary Figures

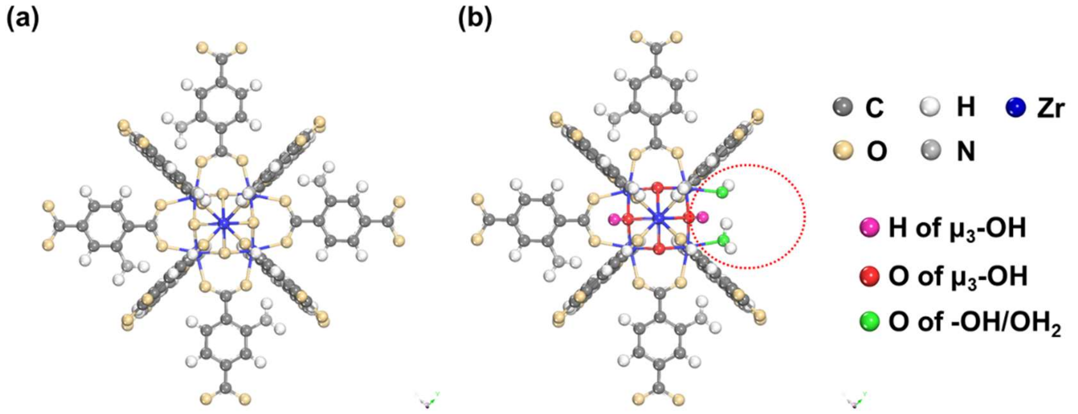

Supplementary Fig. 1 Illustration of the structure of UiO-66-NH_2-(Zr). (a) Zr_6-oxo cluster in ideal UiO-66-NH_2 and (b) Zr_6-oxo cluster in defective UiO-66-NH_2 with terminal -OH/OH_2 groups replacing the linker.

For UiO-66 framework, the structure in Supplementary Fig. 1a is regarded as an ideal structure based on the previous report (7). It has been well documented in many previous studies that the UiO-66 framework with abundant μ_3-OH and terminal -OH/OH_2 groups (Supplementary Fig. 1b) (4, 5, 6) on the Zr_6-oxo node possesses the ability to immobilize extraneous metal ions, including Fe (8, 9), Co (4), Ni (10), Cu (11), and W (12). We intentionally selected this model structure for the immobilization of the Fe atom to investigate the nanoconfinement effects. In particular for Fe, previous studies (8, 9) have confirmed this structure through fitting the XAS spectra by the proposed Fe anchoring model, which are consistent with the data and data fitting in our system (Fig. 2d-2f).

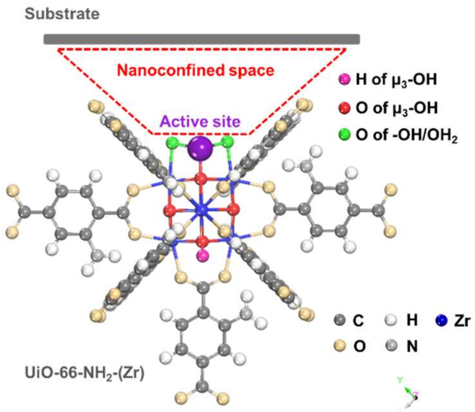

Supplementary Fig. 2 Illustration of the nanoconfined space surrounding the active metal site in UiO-66-NH_2-(Zr) framework.

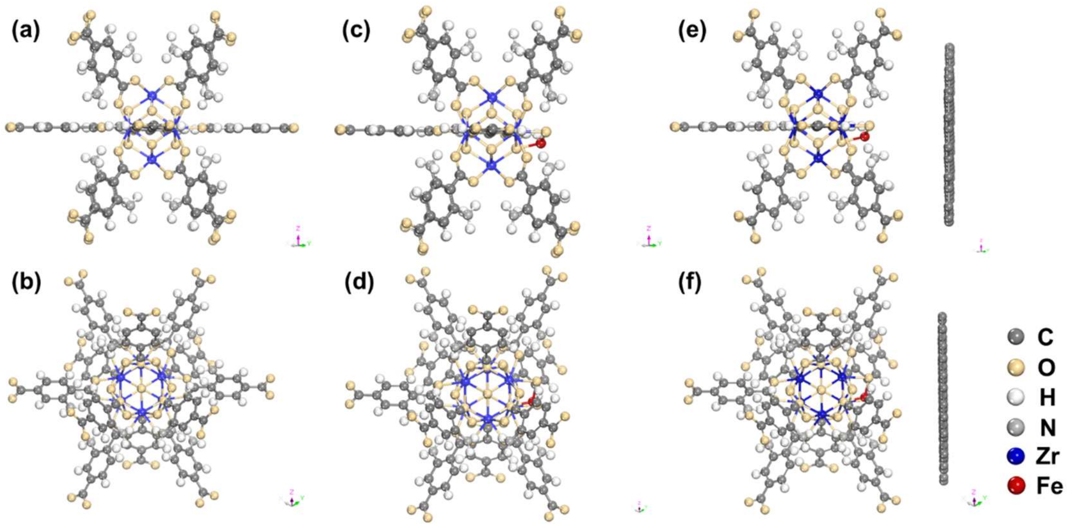

Supplementary Fig. 3 Views of the molecular structures from different angles. (a, b) UiO-66-NH_2-(Zr), (c, d) UiO-66-NH_2-(Zr/Fe), and (e, f) UiO-66-NH_2-(Zr/Fe)/GA.

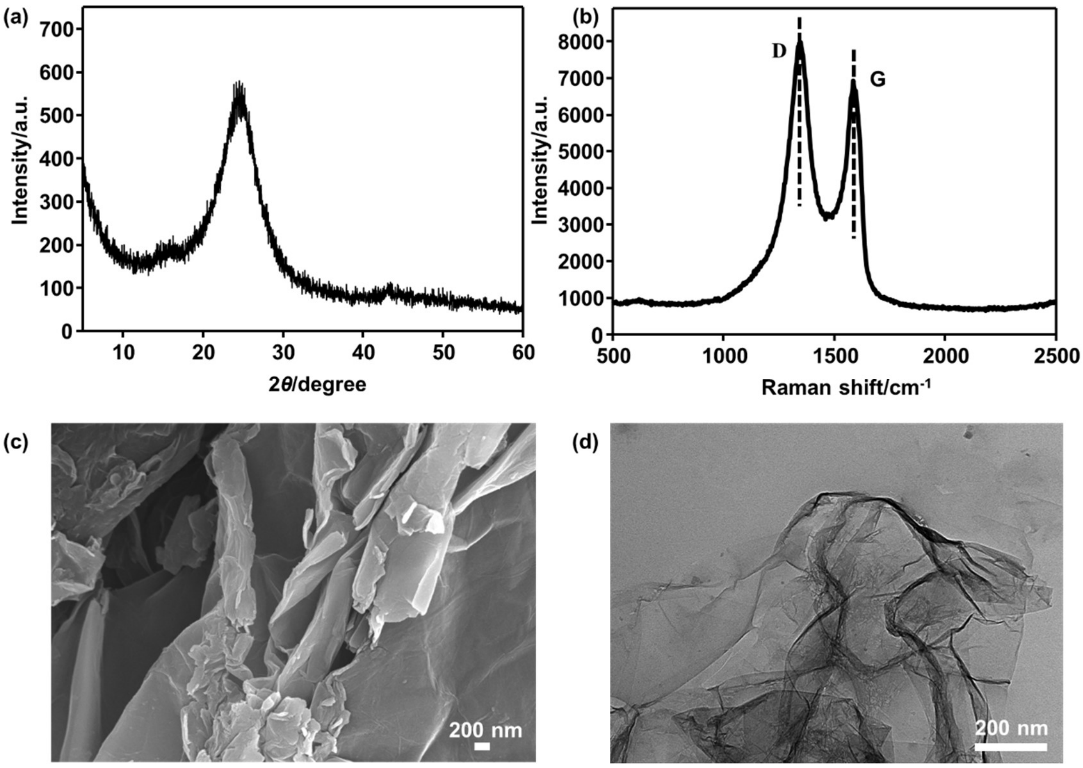

Supplementary Fig. 4 The characterization of GA. Representative (a) XRD patterns, (b) Raman spectra, (c) SEM and (d) TEM images.

The XRD patterns of GA in Supplementary Fig. 4a show a typical broad peak centered at 25° for amorphous reduced graphene oxide sheets. The Raman spectra in Supplementary Fig. 4b exhibit peaks at 1,337 and 1,581 cm^{-1}, corresponding to the characteristic D and G bands of typical carbon materials (13). The nanosheet morphology of the GA substrate is illustrated in the representative SEM and TEM images in Supplementary Fig. 4c and 4d.

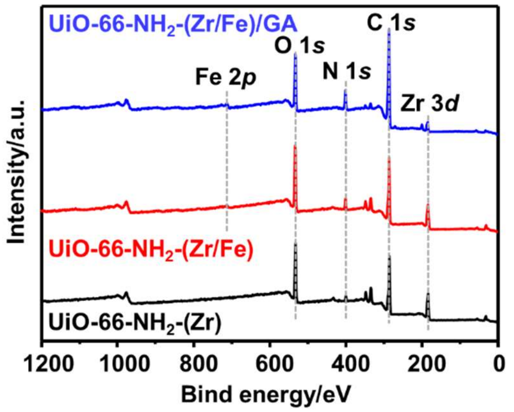

Supplementary Fig. 5 XPS full spectra of UiO-66-NH_2-(Zr), UiO-66-NH_2-(Zr/Fe), and UiO-66-NH_2-(Zr/Fe)/GA.

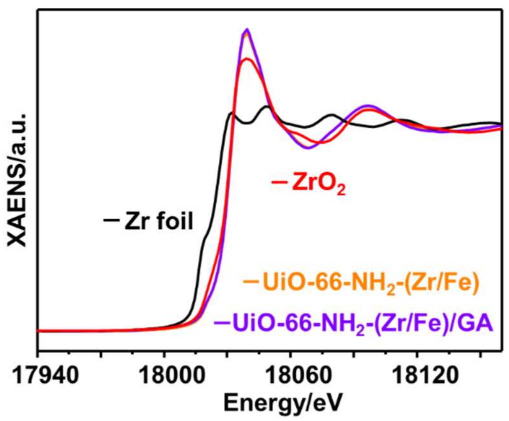

Supplementary Fig. 6 XANES Zr *k*-edge of UiO-66-NH$_2$-(Zr/Fe) and UiO-66-NH$_2$-(Zr/Fe)/GA, using Zr foil and ZrO_2 as references.

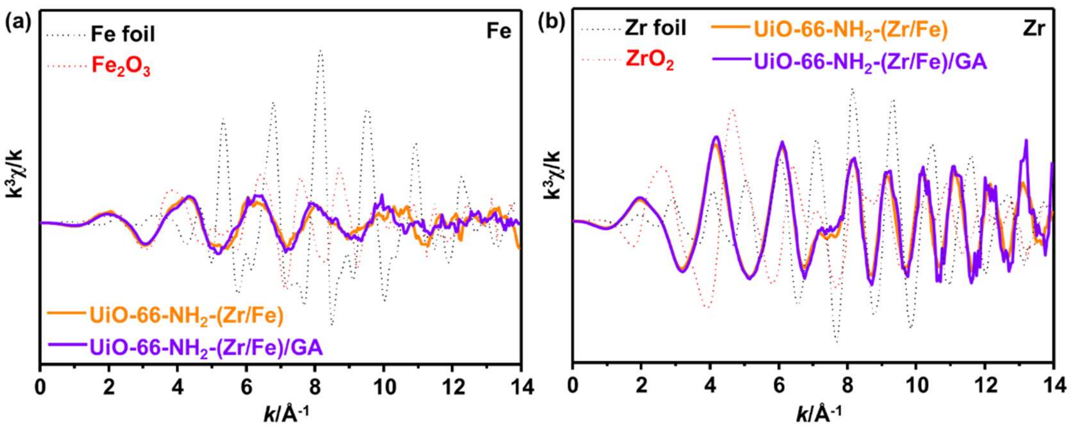

Supplementary Fig. 7 EXAFS signals of UiO-66-NH_2-(Zr/Fe) and UiO-66-NH_2-(Zr/Fe)/GA. (a) Fe *k*-edge signals using Fe foil and Fe_2O_3 as references and (b) Zr *k*-edge signals using Zr foil and ZrO_2 as references.

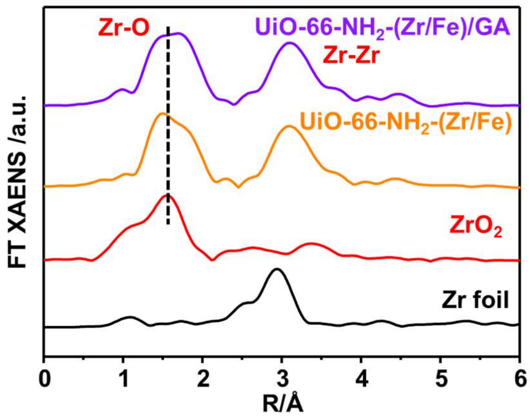

Supplementary Fig. 8 FT-EXAFS Zr *k*-edge spectra in the R space of UiO-66-NH_2-(Zr/Fe) and UiO-66-NH_2-(Zr/Fe)/GA, using Zr foil and ZrO_2 as references.

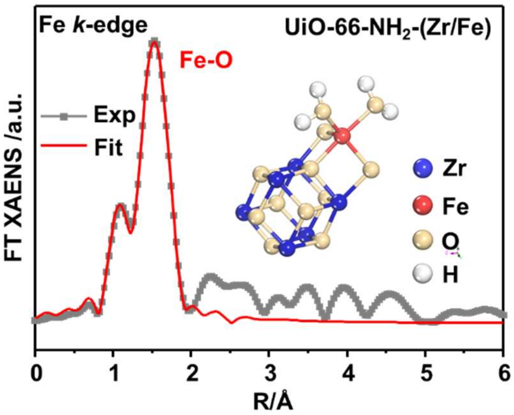

Supplementary Fig. 9 Fitting of the FT-EXAFS Fe *k*-edge curve (inset: fitting structure) of UiO-66-NH_2-(Zr/Fe). The fitting parameters are summarized in Supplementary Table 1.

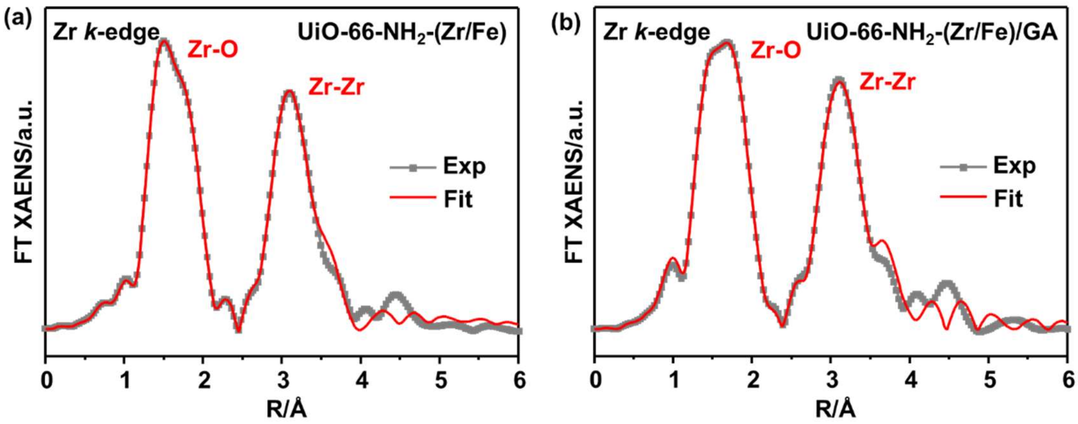

Supplementary Fig. 10 Fitting of the FT-EXAFS Zr *k*-edge curves. (a) UiO-66-NH_2-(Zr/Fe) and (b) UiO-66-NH_2-(Zr/Fe)/GA. The fitting parameters are summarized in Supplementary Table 1.

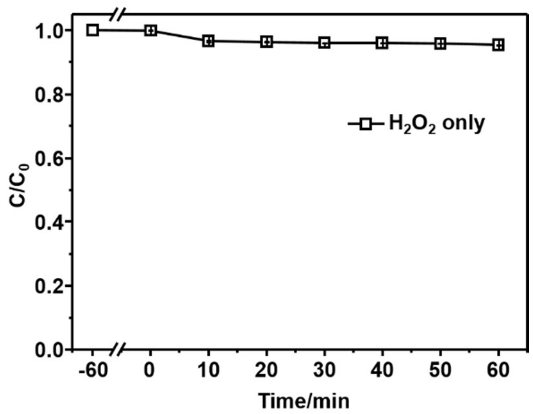

Supplementary Fig. 11 Removal of phenol in H_2O_2 only system. Conditions: initial pH = 5.0 ± 0.2, [H_2O_2] = 6 mM, and [phenol] = 100 μM. The experiments have been carried out in triplicate, and the averaged values with standard deviations as error bars are reported.

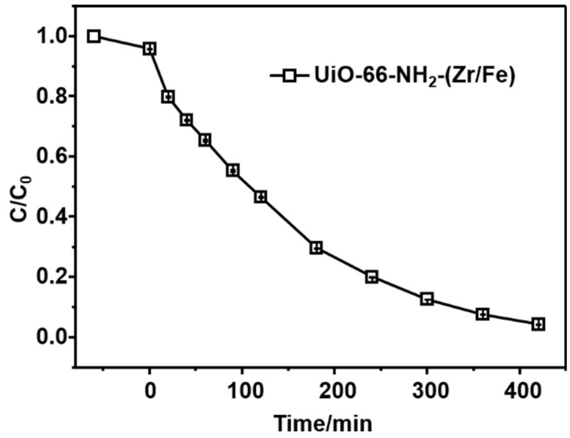

Supplementary Fig. 12 Removal of phenol in UiO-66-NH_2-(Zr/Fe)+H_2O_2 system. Conditions: initial pH = 5.0 ± 0.2, [H_2O_2] = 6 mM, [Catalyst] = 1.0 g·L^{-1}, and [phenol] = 100 μM. The experiments have been carried out in triplicate, and the averaged values with standard deviations as error bars are reported.

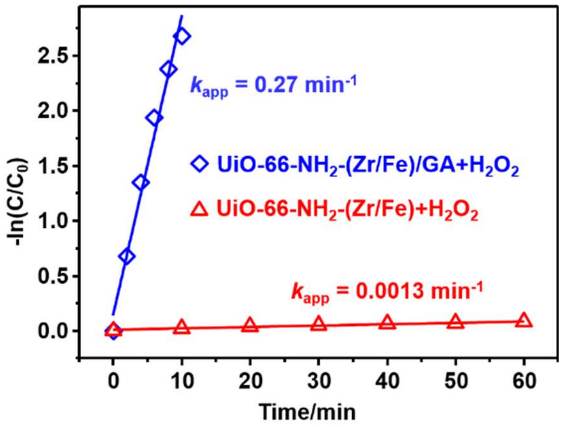

Supplementary Fig. 13 Replotting of the kinetic data for phenol removal in the UiO-66-NH_2-(Zr/Fe)+H_2O_2 and UiO-66-NH_2-(Zr/Fe)/GA+H_2O_2 systems as shown in Fig. 3a, fitted to the first-order reaction kinetics model (solid lines).

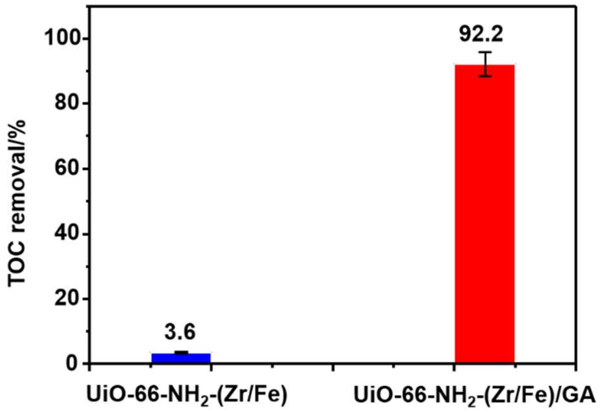

Supplementary Fig. 14 Removal of TOC in 60 min in UiO-66-NH_2-(Zr/Fe)+H_2O_2 and UiO-66-NH_2-(Zr/Fe)/GA+H_2O_2 systems. Conditions: initial pH = 5.0 ± 0.2, [H_2O_2] = 6 mM, [Catalyst] = 100 mg·L^{-1}, and [phenol] = 100 μM. The experiments have been carried out in triplicate, and the averaged values with standard deviations as error bars are reported.

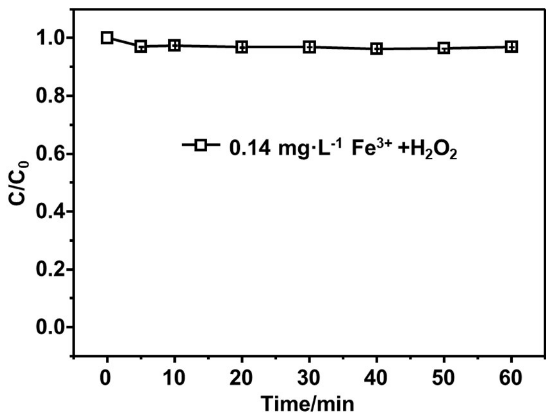

Supplementary Fig. 15 Removal of phenol in Fe^{3+}+H_2O_2 system. Conditions: initial pH = 3.0 ± 0.2, [H_2O_2] = 6 mM, [Fe^{3+}] = 0.14 mg·L^{-1}, and [phenol] = 100 μM. The experiments have been carried out in triplicate, and the averaged values with standard deviations as error bars are reported.

For UiO-66-NH_2-(Zr/Fe)/GA+H_2O_2, the concentration of the dissolved Fe ions after the experiment was determined to be 0.14 mg·L^{-1}, equivalent to 4.8 % of Fe fraction in UiO-66-NH_2-(Zr/Fe)/GA. Supplementary Fig. 15 shows that the contribution of 0.14 mg·L^{-1} Fe ions to the phenol removal is negligible.

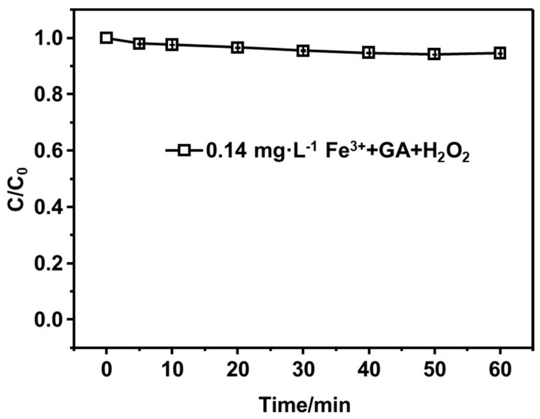

Supplementary Fig. 16 Removal of phenol in Fe^{3+}+GA+H_2O_2 system. Conditions: initial pH = 3.0 ± 0.2, [H_2O_2] = 6 mM, [Fe^{3+}] = 0.14 mg·L^{-1}, [GA] = 100 mg·L^{-1}, and [phenol] = 100 μM. The experiments have been carried out in triplicate, and the averaged values with standard deviations as error bars are reported.

Supplementary Fig. 16 shows that the Fe^{3+}+GA+H_2O_2 system makes negligible contribution to the phenol removal, i.e., only 4.4 % removal in 60 min, ruling out the contribution of the leached Fe ions + GA for phenol removal.

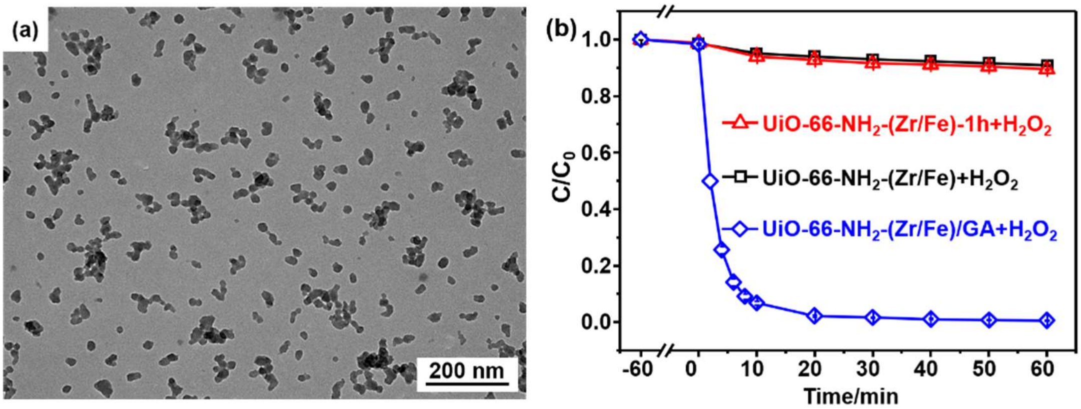

Supplementary Fig. 17 The characterization of UiO-66-NH_2-(Zr/Fe)-1h and its catalytic performance. (a) Representative TEM image and (b) Removal of phenol compared to UiO-66-NH_2-(Zr/Fe)+H_2O_2 and UiO-66-NH_2-(Zr/Fe)/GA+H_2O_2. Conditions: initial pH = 5.0 ± 0.2, [H_2O_2] = 6 mM, [Catalyst] = 100 mg·L^{-1}, and [phenol] = 100 μM. The experiments have been carried out in triplicate, and the averaged values with standard deviations as error bars are reported.

The UiO-66-NH_2-(Zr/Fe)-1h sample was prepared using the hydrothermal time of only one hour, which is much shorter than that (24 hours) for UiO-66-NH_2-(Zr/Fe) and UiO-66-NH_2-(Zr/Fe)/GA, with other parameters identical. Supplementary Fig. 17a shows that the UiO-66-NH_2-(Zr/Fe)-1h contains particles with irregular shape and size of only around 40 nm, which is much smaller than that in UiO-66-NH_2-(Zr/Fe) (Fig. 1c and 1d) and similar to that in UiO-66-NH_2-(Zr/Fe)/GA (Fig. 1e and 1f). Supplementary Fig. 17b shows that the UiO-66-NH_2-(Zr/Fe)-1h+H_2O_2 system exhibits similar catalytic reactivity towards phenol removal to the UiO-66-NH_2-(Zr/Fe)+H_2O_2 system, both are much lower than the UiO-66-NH_2-(Zr/Fe)/GA+H_2O_2 system. These results exclude the possible influence of MOFs particle size on the dramatic difference of their reactivity.

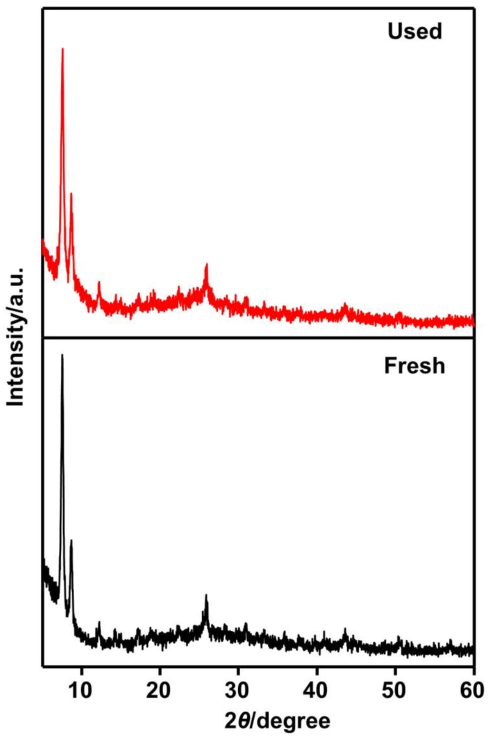

Supplementary Fig. 18 XRD patterns of the UiO-66-NH_2-(Zr/Fe)/GA catalyst before (Fresh) and after (Used) the catalytic experiments.

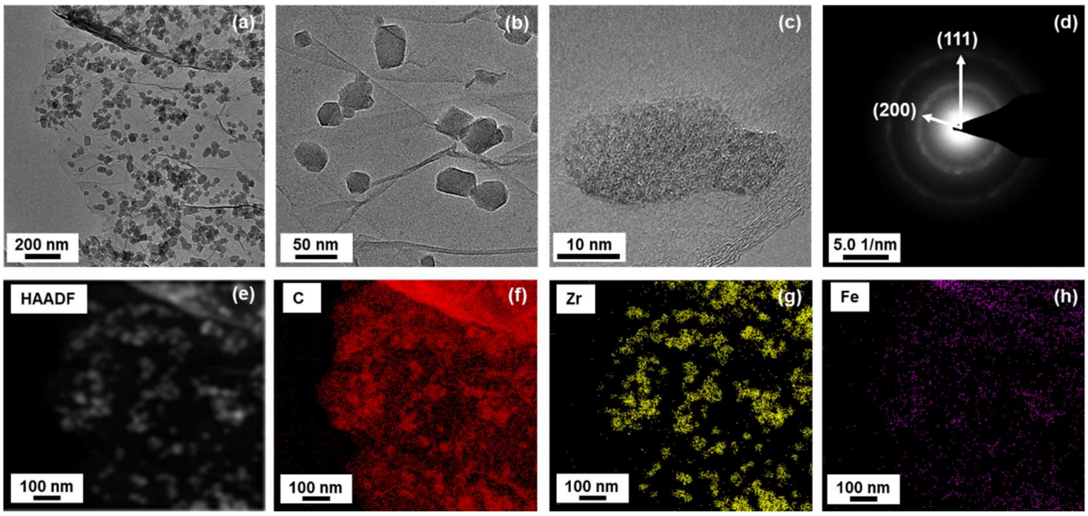

Supplementary Fig. 19 The electron microscope characterization of used UiO-66-NH_2-(Zr/Fe)/GA sample. (a) TEM image, (b) and (c) HRTEM images, (d) SAED patterns, and (e-h) HAADF and EDS elemental mapping images.

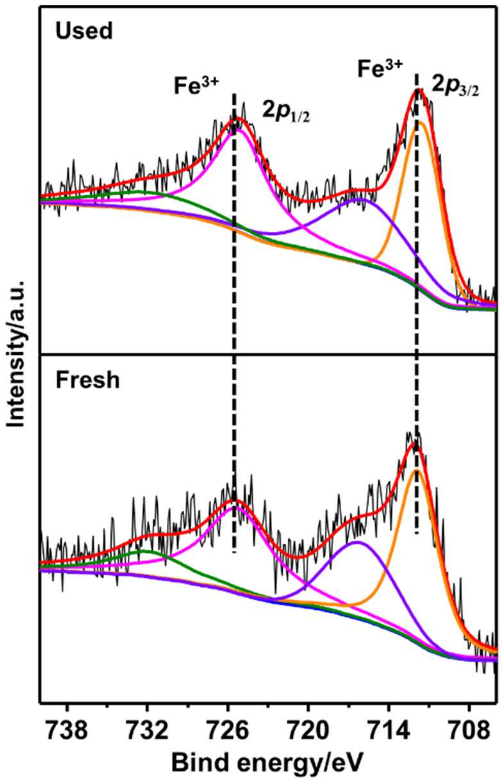

Supplementary Fig. 20 The deconvolutions of Fe 2*p* XPS spectra of the UiO-66-NH_2-(Zr/Fe)/GA catalyst before (Fresh) and after (Used) the catalytic experiments.

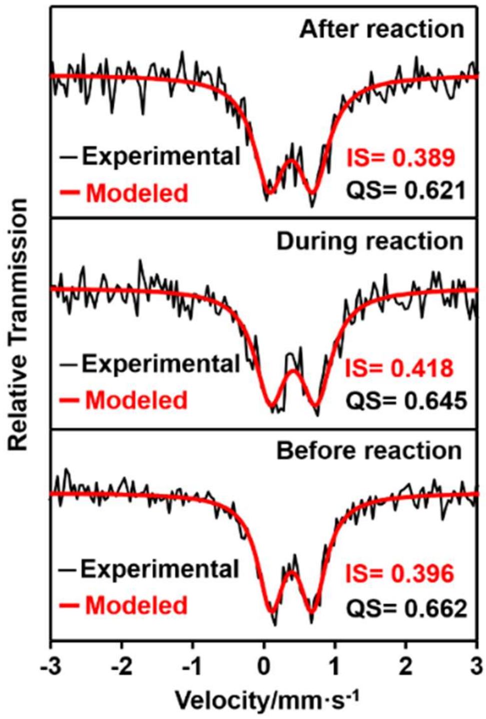

Supplementary Fig. 21 ^{57}Fe Mössbauer spectra of the UiO-66-NH_2-(Zr/Fe)/GA samples before, during, and after the catalytic reaction at 25°C.

Supplementary Fig. 21 shows that there is an increase of the electron density of Fe (an increase of IS value) during reaction. After the reaction, the electron density of Fe decreases back to the initial state (a decrease of the IS value). These results are consistent with the reaction pathways as proposed in Fig. 4.

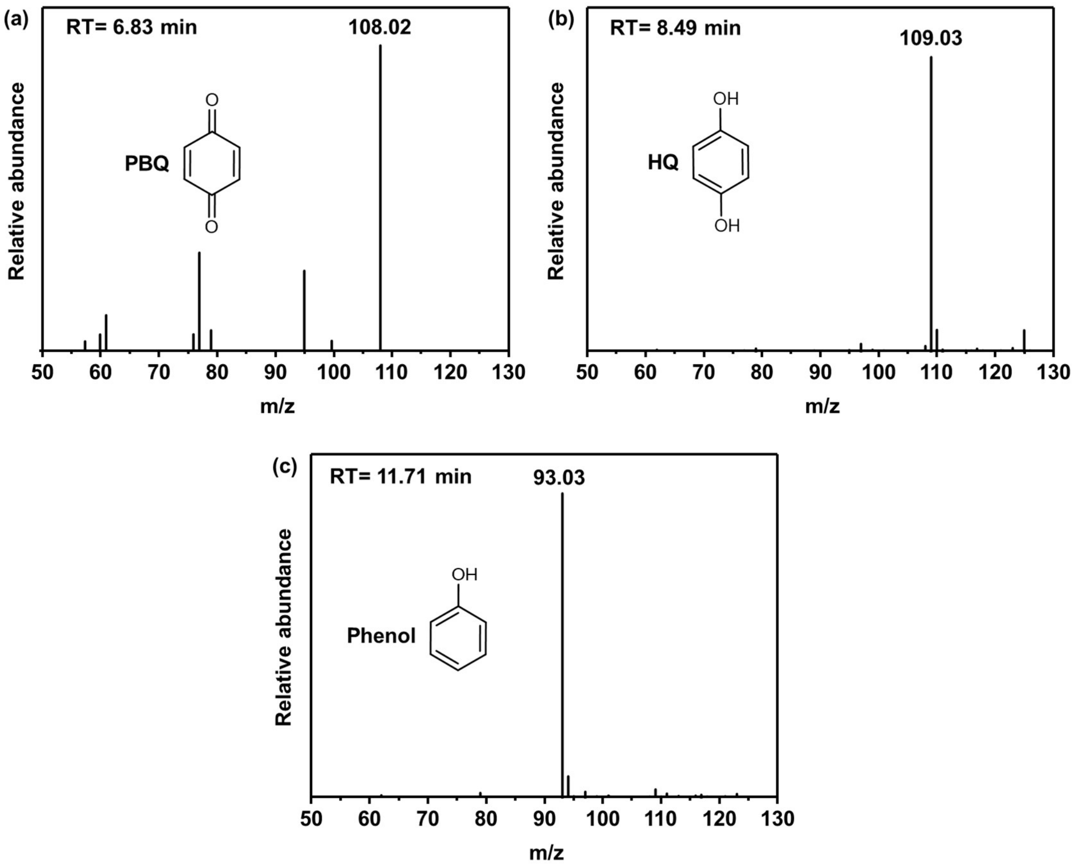

Supplementary Fig. 22 UHPLC-MS spectra of the intermediates detected at different retention time (RT) for phenol removal in the UiO-66-NH_2-(Zr/Fe)+H_2O_2 system at reaction time of 60 min (14, 15). (a) 6.83 min, PBQ, (b) 8.49 min, HQ, and (c) 11.71 min, phenol.

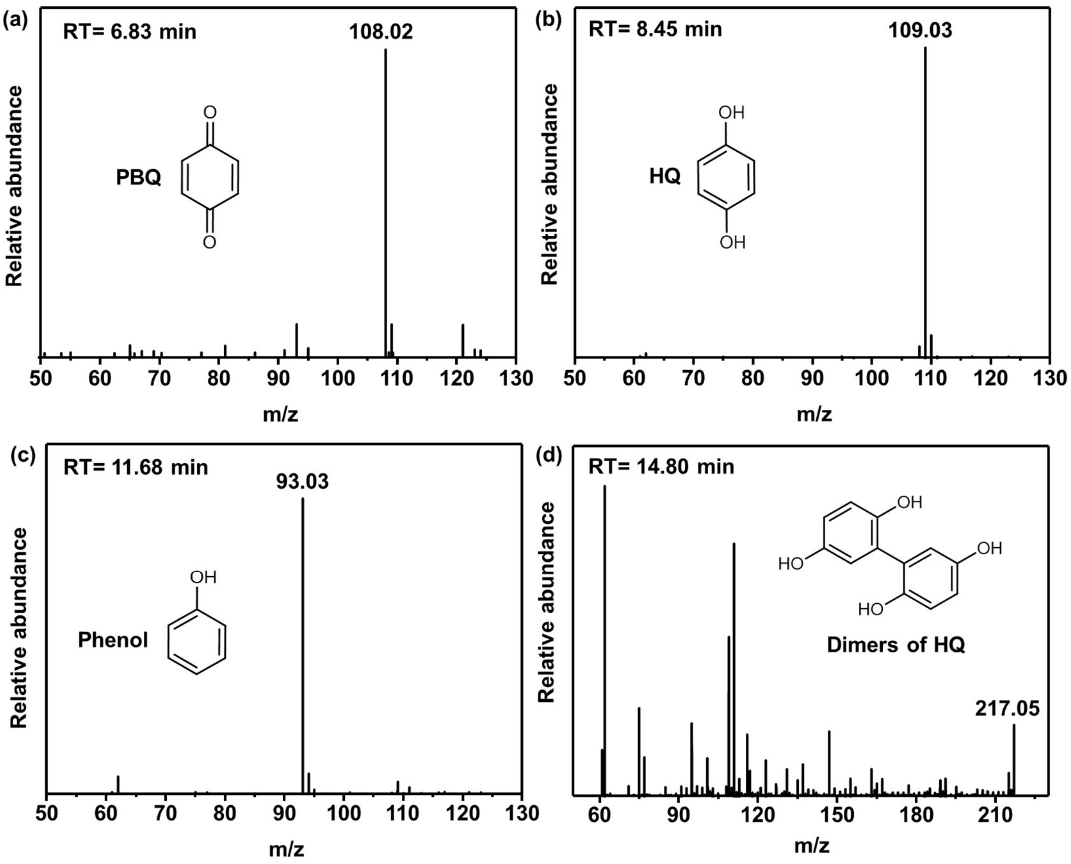

Supplementary Fig. 23 UHPLC-MS spectra of the intermediates detected at RT for phenol removal in the UiO-66-NH_2-(Zr/Fe)/GA+H_2O_2 system at reaction time of 10 min (14, 15). (a) 6.83 min, PBQ, (b) 8.45 min, HQ, (c) 11.68 min, phenol, and (d) 14.80 min, dimers of HQ.

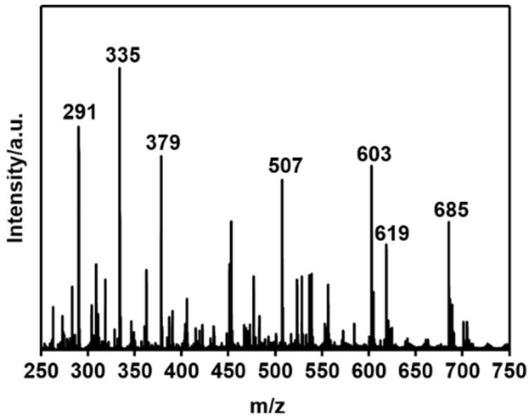

Supplementary Fig. 24 MALDI-TOF MS signals of the products obtained by washing the used UiO-66-NH_2-(Zr/Fe)/GA catalyst by THF. The proposed structures of the oligomers with marked m/z values are listed in Supplementary Table 3.

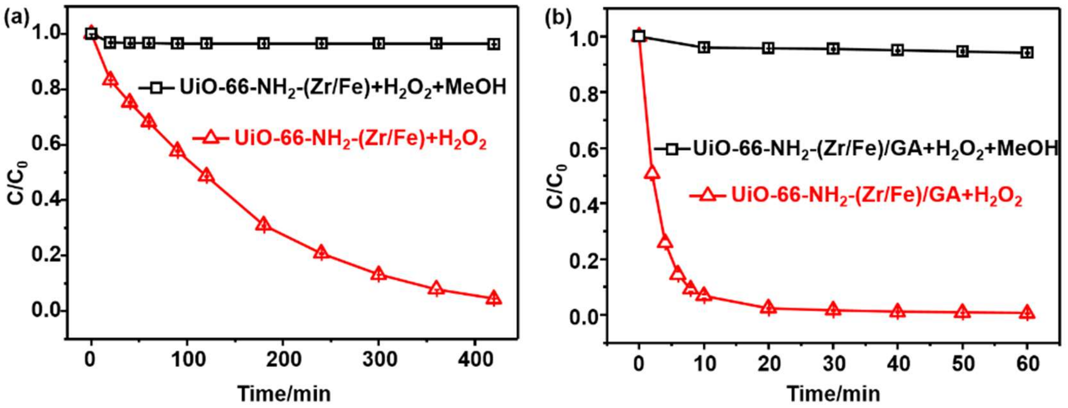

Supplementary Fig. 25 Removal of phenol without and with excessive MeOH (1.0 M). (a) UiO-66-NH_2-(Zr/Fe)+H_2O_2 and (b) UiO-66-NH_2-(Zr/Fe)/GA+H_2O_2. Conditions: initial pH = 5.0 ± 0.2, [H_2O_2] = 6 mM, [Catalyst] = 1.0 g·L^{-1} for (a), [Catalyst] = 100 mg·L^{-1} for (b), and [phenol] = 100 μM. The experiments have been carried out in triplicate, and the averaged values with standard deviations as error bars are reported.

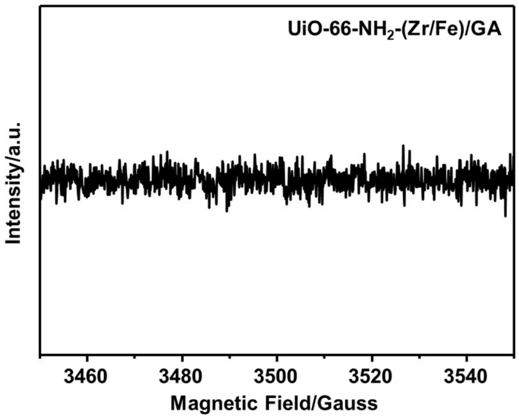

Supplementary Fig. 26 EPR signals by using TEMP as probing molecule in the UiO-66-NH_2-(Zr/Fe)/GA+H_2O_2 system at the reaction time of 5 min. Conditions: initial pH = 5.0 ± 0.2, [H_2O_2] = 6 mM, [Catalyst] = 100 mg·L^{-1}, and [phenol] = 100 μM.

Supplementary Fig. 26 shows that $^1O_2$ is not generated in the UiO-66-NH_2-(Zr/Fe)/GA+H_2O_2 system, because the addition of $^1O_2$ trapping agent, i.e., TEMP, does not exhibit any signals.

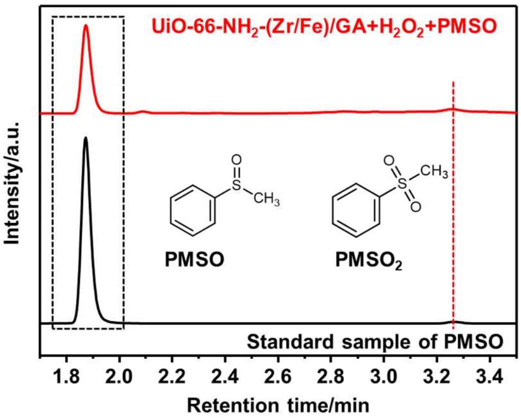

Supplementary Fig. 27 Transformation of PMSO by UiO-66-NH$_2$-(Zr/Fe)/GA+H_2O_2. Conditions: initial pH = 5.0 ± 0.2, [H_2O_2] = 6 mM, [Catalyst] = 100 mg·L^{-1}, and [PMSO] = 100 μM.

Supplementary Fig. 27 shows that high-valent Fe is not generated in the UiO-66-NH$_2$-(Zr/Fe)/GA+H_2O_2 system, because the unique transformation from PMSO to PMSO$_2$ by high-valent Fe is not observed.

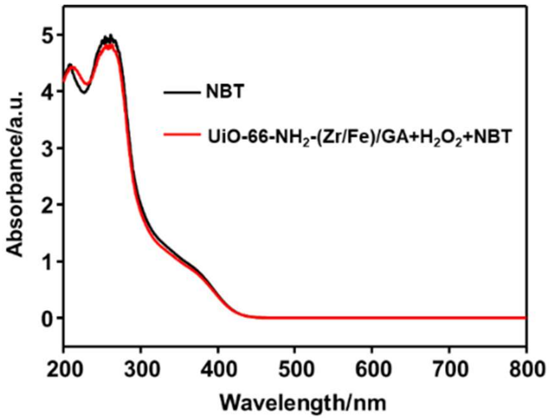

Supplementary Fig. 28 UV-vis adsorption of NBT solution in UiO-66-NH$_2$-(Zr/Fe)/GA+H_2O_2 system. Conditions: initial pH = 5.0 ± 0.2, [H_2O_2] = 6 mM, [Catalyst] = 100 mg·L^{-1}, and [NBT] = 100 μM.

Supplementary Fig. 28 shows that $O_2^{\bullet-}$ is not generated the UiO-66-NH$_2$-(Zr/Fe)/GA+H_2O_2 system because if $O_2^{\bullet-}$ is generated, the addition of NBT as the chemical probe should be reduced by $O_2^{\bullet-}$ to form monoformazan which exhibits a maximum absorption at 530 nm (16).

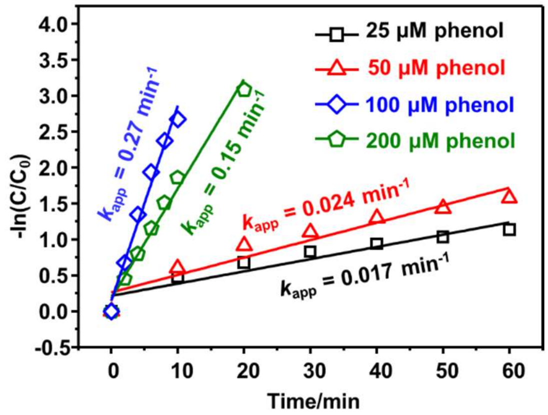

Supplementary Fig. 29 Replot of data in Fig. 3e fitted to the pseudo first-order reaction kinetics model (solid lines). Reaction conditions: pH = 5.0 ± 0.2, [H_2O_2] = 6 mM, [Catalyst] = 100 mg·L^{-1}, and [phenol] = 25-200 μM.

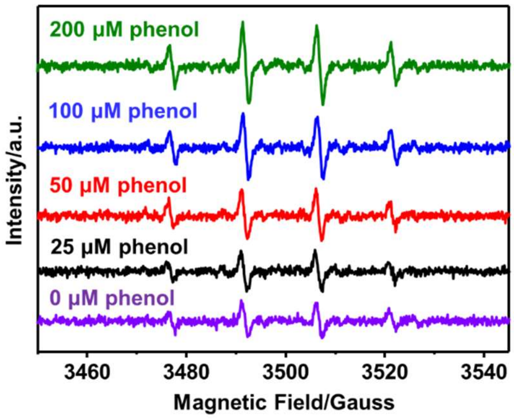

Supplementary Fig. 30 EPR signals by using DMPO as probing molecule in the UiO-66-NH_2-(Zr/Fe)/GA+H_2O_2 system with different phenol initial concentrations at the same reaction time (5 min). Conditions: initial pH = 5.0 ± 0.2, [H_2O_2] = 6 mM, [Catalyst] = 100 mg·L^{-1}, and [phenol] = 25-200 μM.

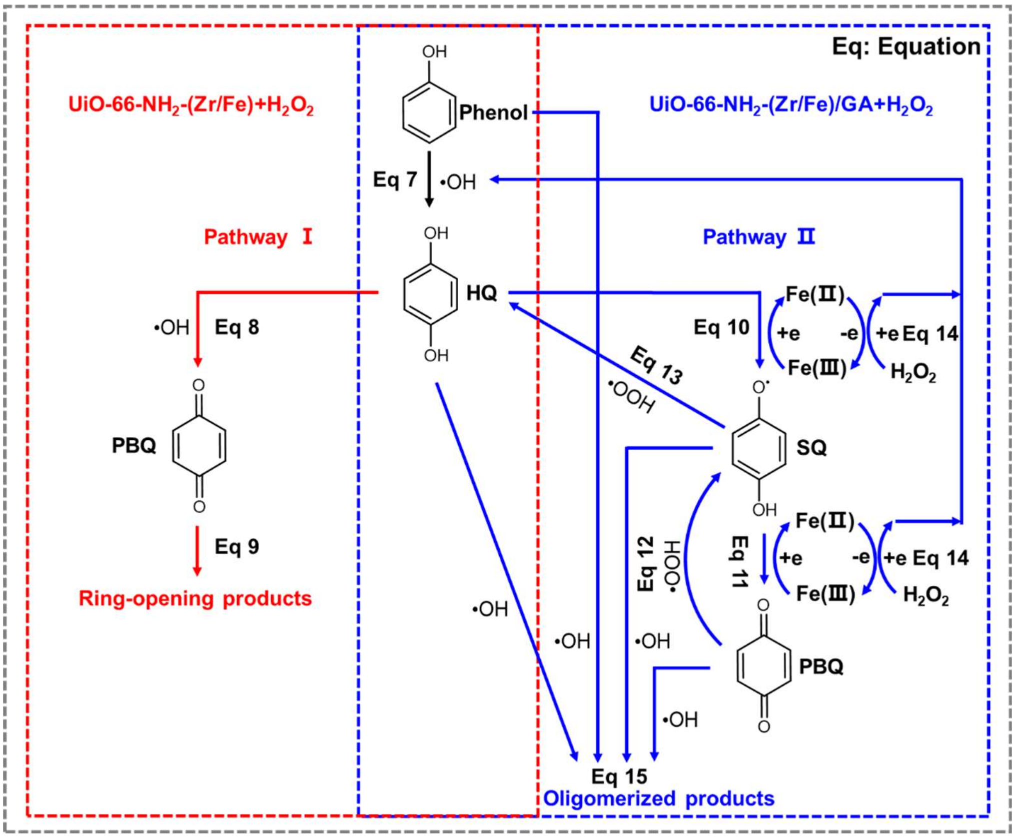

Supplementary Fig. 31 Proposed phenol removal pathways in UiO-66-NH$_2$-(Zr/Fe)+H$_2$O$_2$ and UiO-66-NH$_2$-(Zr/Fe)/GA+H$_2$O$_2$ systems, with the key reactions marked.

The key reactions involved in both Pathway I and II are:

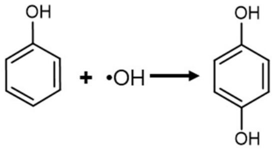

$k_7 = 7.3 \times 10^9$ M^{-1}s^{-1}(17) Supplementary Equation (7)

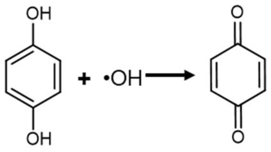

$k_8 = {\sim}10^9$ M^{-1}s^{-1}(17, 18) Supplementary Equation (8)

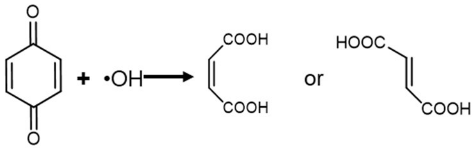

$k_9 = {\sim}10^9$ M^{-1}s^{-1}(17) Supplementary Equation (9)

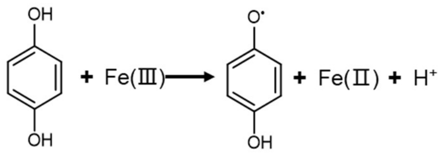

$k_{10} = 4.4 \times 10^2\ M^{-1}s^{-1}$(19, 20, 21) Supplementary Equation (10)

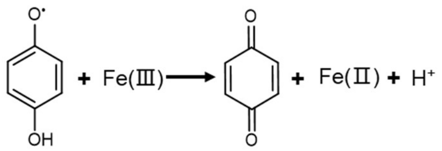

$k_{11} = 4.4 \times 10^4\ M^{-1}s^{-1}$(19, 20, 21) Supplementary Equation (11)

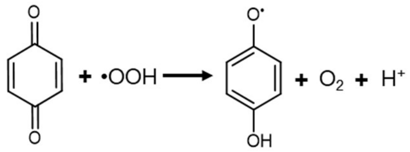

$k_{12} = 1 \times 10^9\ M^{-1}s^{-1}$(19, 20, 21) Supplementary Equation (12)

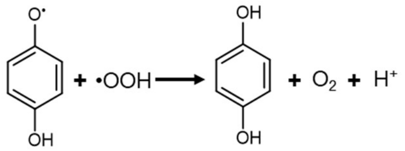

$k_{13} = 1 \times 10^9\ M^{-1}s^{-1}$(19, 20, 21) Supplementary Equation (13)

$$Fe(II) + H_2O_2 \longrightarrow Fe(III) + {\bullet}OH + OH^-$$

$k_{14} = 76\ M^{-1}s^{-1}$(19, 20, 21) Supplementary Equation (14)

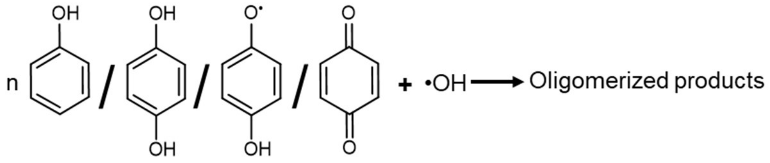

(17, 22, 23) Supplementary Equation (15)

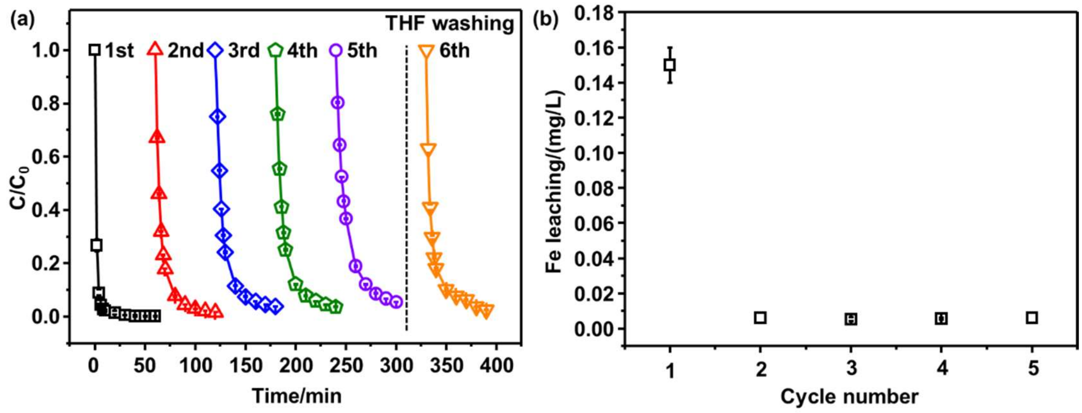

Supplementary Fig. 32 The cyclic experiments of UiO-66-NH_2-(Zr/Fe)/GA. (a) Six consecutive phenol removal. (b) The concentration of leaching Fe in the solution after the reaction in UiO-66-NH_2-(Zr/Fe)/GA+H_2O_2 system in the first five consecutive run. Conditions: initial pH = 4.0 ± 0.2, [H_2O_2] = 6 mM, [Catalyst] = 100 mg·L^{-1}, and [phenol] = 100 μM. In the first five runs, the catalyst was washed by water three times between each run. After five runs, the catalyst was washed by THF for the sixth run. The experiments have been carried out in triplicate, and the averaged values with standard deviations as error bars are reported.

Supplementary Fig. 32a shows that in the first five consecutive runs where the catalyst was washed by water, there is a gradual decrease of the reactivity for phenol removal. The corresponding Fe leaching in each run is shown in Supplementary. Fig. 32b, showing a ca. 0.15 mg·L^{-1} Fe leaching (5.0 % Fe mass in the catalyst) in the first run and negligible Fe leaching in the following runs. Such decrease of reactivity is presumably due to the accumulation of oligomerized products that block the catalytic sites. A further THF washing treatment of the catalyst could recover its reactivity effectively, as shown in the sixth run.

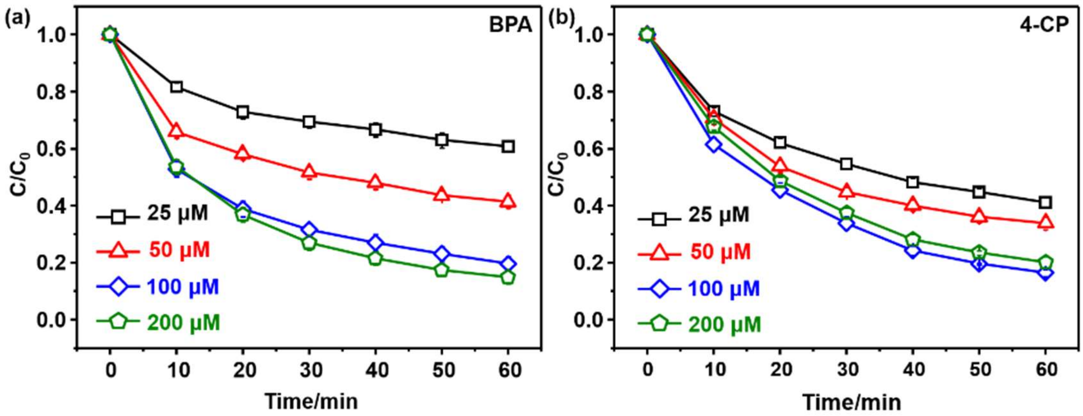

Supplementary Fig. 33 Degradation of various phenolic pollutants with different initial concentration by UiO-66-NH_2-(Zr/Fe)/GA+H_2O_2 system. (a) BPA and (b) 4-CP. Conditions: pH = 5.0 ± 0.2, [H_2O_2] = 6 mM, [Catalyst] = 100 mg·L^{-1}, and [Pollutant] = 25-200 μM. The experiments have been carried out in triplicate, and the averaged values with standard deviations as error bars are reported.

Results in Supplementary Fig. 33 show that the removal of BPA and 4-CP by the UiO-66-NH_2-(Zr/Fe)/GA+H_2O_2 system exhibits similar behaviors to that of phenol, i.e., accelerated removal as the increase of the pollutant initial concentration. Previous studies have confirmed that the oxidation of both BPA and 4-CP could generate quinone-like intermediates (24, 25), which should allow the Pathway II removal pathway as proposed. Moreover, it is noticed that the removal rates for BPA and 4-CP in Fig. 33 are apparently lower than that for phenol, presumably, because it is more difficult for BPA and 4-CP to transform to quinone-like intermediates than phenol due to the substitutional groups on the benzene ring.

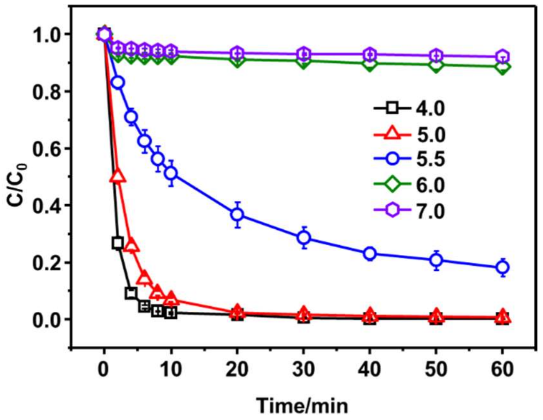

Supplementary Fig. 34 Removal of phenol by the UiO-66-NH_2-(Zr/Fe)/GA+H_2O_2 system at different initial pH. Conditions: [H_2O_2] = 6 mM, [Catalyst] = 100 mg·L^{-1}, and [phenol] = 100 μM. The experiments have been carried out in triplicate, and the averaged values with standard deviations as error bars are reported.

Supplementary Fig. 34 shows that the UiO-66-NH_2-(Zr/Fe)/GA+H_2O_2 system exhibits excellent phenol removal at initial pH of 4.0 and 5.0. When the initial pH is further increased to 5.5, the removal decreases obviously, 63.2% in 20 min and 81.9% in 60 min. The catalytic system can hardly remove phenol when the initial pH is increased to 6.0 and 7.0. The reduction of removal efficiency is apparently due to the reduced catalytic reactivity of Fe atoms in heterogeneous Fenton reactions under elevated pH conditions and reduced activity of •OH (25, 26, 27).

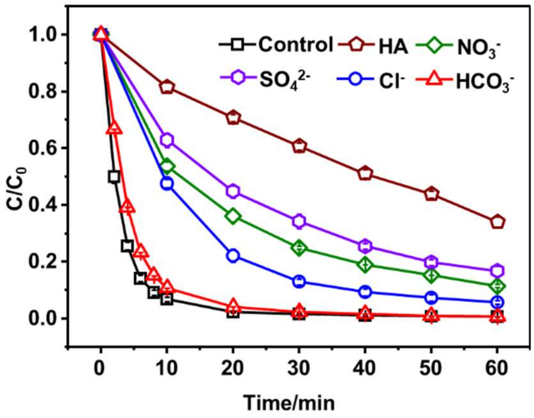

Supplementary Fig. 35 Removal of phenol by UiO-66-NH_2-(Zr/Fe)/GA/H_2O_2 (blank) in the presence of various coexisting ions, i.e., NO_3^- (10 mM), SO_4^{2-} (10 mM), Cl^- (10 mM), HCO_3^-(10 mM), and HA(10 mg·L^{-1}). Conditions: initial pH = 5.0 ± 0.2, [H_2O_2] = 6 mM, [Catalyst] = 100 mg·L^{-1}, and [phenol] = 100 μM. The experiments have been carried out in triplicate, and the averaged values with standard deviations as error bars are reported.

Supplementary Fig. 35 shows that HCO_3^- brings insignificant effects on the phenol removal, while Cl^-, SO_4^{2-}, and NO_3^- inhibits the phenol removal to different extents. These results are consistent with other •OH-mediated systems reported previously (28, 29), presumably due to the reaction between •OH and these anions. Moreover, the presence of natural organic substance HA greatly suppresses the phenol removal because of the competitive consumption of the •OH reactive species by HA (30).

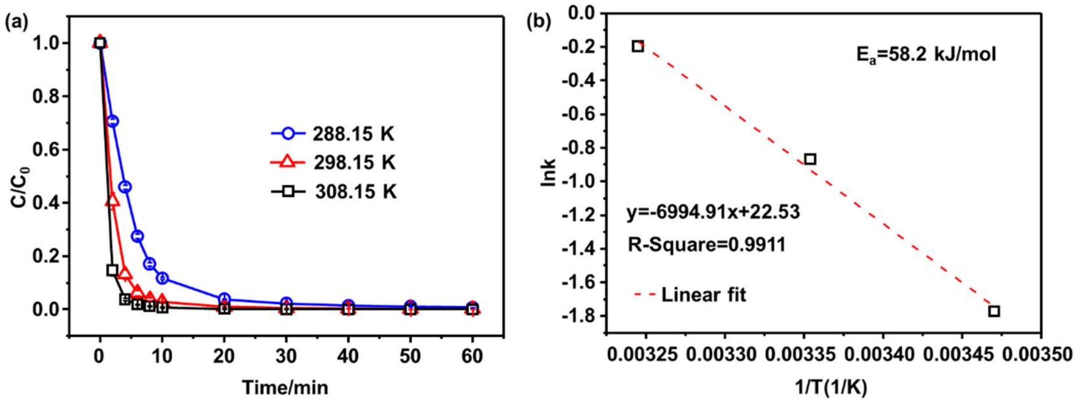

Supplementary Fig. 36 The effect of reaction temperature on phenol removal by the UiO-66-NH_2-(Zr/Fe)/GA+H_2O_2 system. (a) Removal of phenol at different temperatures and (b) the Arrhenius curve of phenol degradation at different temperatures. The experiments have been carried out in triplicate, and the averaged values with standard deviations as error bars are reported.

Supplementary Fig. 36a shows that the phenol removal by the UiO-66-NH_2-(Zr/Fe)/GA+H_2O_2 system is affected by the reaction temperature. Based on the first-order reaction rate constant at different temperatures, we could build the Arrhenius plot in Supplementary Fig. 36b, from which the activation energy of the reaction is calculated to be 58.2 kJ·mol^{-1}.

Supplementary Tables

Supplementary Table 1. Structural parameters of UiO-66-NH_2-(Zr/Fe) and UiO-66-NH_2-(Zr/Fe)/GA samples extracted from the EXAFS data fitting.

Samples	Shell	R (Å)[a]	CN[b]	σ^2 (Å^2*10^{-3})[c]	ΔE_0 (eV)[d]	R-factor[e]
UiO-66(Fe/Zr)-NH_2	Zr-O1	2.15	4.5	3.9±0.8	2.4±0.9	0.001
	Zr-O2	2.28	3.5	3.8±0.5	9.3±0.7	
UiO-66(Fe/Zr)-NH_2/GA	Zr-O1	1.98	4.0	4.4±0.4	9.2±0.8	0.001
	Zr-O2	2.27	3.8	6.3±0.7	1.5±0.9	
UiO-66(Fe/Zr)-NH_2	Fe-O1	1.72	2.8	4.8±0.7	1.4±0.3	0.026
	Fe-O2	2.00	2.1	8.7±0.8	1.9±0.4	
UiO-66(Fe/Zr)-NH_2/GA	Fe-O1	1.75	2.7	9.4±0.6	4.7±0.7	0.006
	Fe-O2	2.03	1.9	2.1±0.5	6.7±0.5	

[a] R: bond distance; [b] CN: coordination numbers; [c] σ^2: Debye-Waller factors; [d] ΔE_0: the inner potential correction; [e] R-factor: goodness of fit.

Supplementary Table 2. GC-MS chromatograms of the products (after derivation by TMSCl) generated in the phenol degradation by UiO-66-NH_2-(Zr/Fe)+H_2O_2.

Retention time (min)	Product [a]	m/z	Molecular formula	Molecular structure
4.0	Acetic acid	60	$C_2H_4O_2$	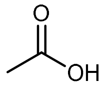
3.3	Oxalic acid	90	$C_2H_2O_4$	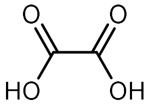
11.6	Malonic acid	104	$C_3H_4O_4$	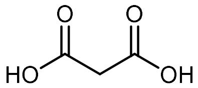

[a] The products are determined by the GC-MS analysis.

Supplementary Table 3. Proposed structures based on the MALDI-TOF MS spectra in Supplementary Fig. 24.

Products	m/z	Relative molecular mass	Molecular formula	Proposed structure[a],[b]
P1	291	290	$C_{15}H_{14}O_6$	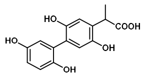
P2	335	334	$C_{16}H_{14}O_8$	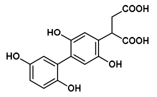
P3	379	378	$C_{21}H_{14}O_7$	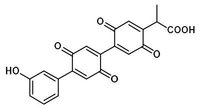
P4	507	506	$C_{30}H_{18}O_8$	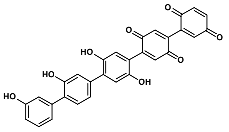
P5	603	602	$C_{36}H_{26}O_9$	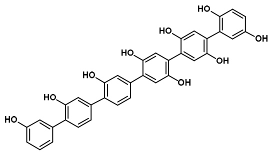
P6	619	618	$C_{36}H_{26}O_{10}$	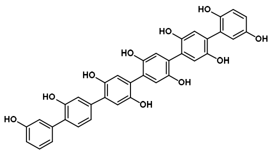
P7	685	684	$C_{40}H_{28}O_{11}$	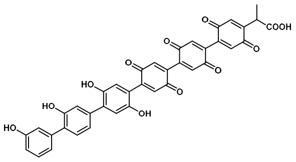

[a] The structures are proposed based on the possible monomers including phenol, HQ, and PBQ, and the reported Fenton degradation reaction path (17, 18).

[b] There might be multiple isomers for the same chemical formulas, and only one of them is proposed in this table.

Supplementary Table 4. H_2O_2 consumption for TOC removal of phenol solution by homogeneous Fe^{2+}+H_2O_2 systems and heterogeneous UiO-66-NH_2-(Zr/Fe)+H_2O_2 system.

Catalyst	Initial H_2O_2 concentration (mM)	H_2O_2 consumption (mM)	TOC removal (%)	TOC removal ($mg \cdot L^{-1}$)	H_2O_2 consumption/TOC removal ($mmol \cdot mg^{-1}$)
0.25 mM Fe^{2+}	6	4.74	29.5	2.04	2.26
0.25 mM Fe^{2+}	12	10.0	23.1	1.60	6.25
0.5 mM Fe^{2+}	6	5.36	35.5	2.73	1.93
0.5 mM Fe^{2+}	12	10.3	33.5	2.58	3.99
100 $mg \cdot L^{-1}$ UiO-66-NH_2-(Zr/Fe)/GA	6	0.532	92.2	6.09	0.0874

Conditions: initial pH = 3.0 ± 0.2 for homogeneous Fenton systems, initial pH = 5.0 ± 0.2 for UiO-66-NH_2-(Zr/Fe)+H_2O_2 system, [phenol] = 100 μM, Reaction time = 60 min.

Note that the homogeneous Fe^{2+}+H_2O_2 system is normally less effective for high degree mineralization, which is consistent with previous studies (17, 31). Consequently, we use the ratio of H_2O_2 consumption to TOC removal to compare the H_2O_2 efficiency in various systems. Based on the results in Supplementary Table 4, the heterogeneous UiO-66-NH_2-(Zr/Fe)+H_2O_2 system could reduce the H_2O_2 usage by more than 95 % for TOC removal, compared to the homogeneous Fe^{2+}+H_2O_2 systems.

Supplementary Table 5. Comparison of the experimental parameters in related heterogeneous Fenton reactions.

Catalysts	Dosage of Catalyst ($mg\cdot L^{-1}$)	H_2O_2 (mM)	Contaminant, (mM)	Ratio of H_2O_2/Contaminant	Initial pH	TOC removal	Ref.
MIL-101-(Fe)	100	6	phenol, 0.10	60	5.0	13.6% in 1 h 25℃	(27)
GA/MIL-101-(Fe)	100	6	phenol, 0.10	60	5.0	33.5% in 1 h 25℃	(27)
FeCu/Al-containing MCM-41	1500	49	phenol, 2.12	23	4.0	47% in 2 h 60℃	(32)
Fe-Al-MPM	500	10	phenol, 0.53	19	3.0	78 % in 4 h 30℃	(33)
Iron-carbon	3000	11.8	phenol, 1.06	11	3.0	68.2% in 2.5 h 60℃	(34)
K-FeOCl	1000	50	2-MeOP[a], 0.08	625	7.0	100% in 4 h 25 ℃	(35)
Cu5/FeS_2	500	0.8	Alachlor, 0.04	20	7.0	49.5% in 1.5 h Ambient temperature	(36)
Fe_2O_3/FCNT-L	15	50	MB[b], 0.001	5000	5.0	24% in 1 h 20 ℃	(37)
UiO-66-NH_2-(Zr/Fe)	100	6	phenol, 0.10	60	5.0	3.6% in 1 h 25℃	This work
UiO-66-NH_2-(Zr/Fe)/GA	100	6	phenol, 0.10	60	5.0	92.2% in 1 h 25℃	This work

[a] 2-MeOP represents guaiacol, [b] MB represents methylene blue

Supplementary references

1. Ravel, B. & Newville. M. ATHENA, ARTEMIS, HEPHAESTUS: data analysis for X-ray absorption spectroscopy using IFEFFIT. *J. Synchrotron Radiat.* **12**, 537-541 (2005).

2. Koningsberger, D. C. & Prins. R. X-ray Absorption: Principles, Applications, Techniques of EXAFS, SEXAFS, and XANES (eds Koningsberger, D. C.; Prins, R.) (Wiley, 1988), 92.

3. Rehr, J. J. & Albers, R. C. *Rev. Mod. Phys.* **72**, 621-654 (2000).

4. Manna, K. et al. Chemoselective single-site earth-abundant metal catalysts at metal-organic framework nodes. *Nat. Commun.* **7**, 12610 (2016).

5. Islamoglu, T. et al. Postsynthetic tuning of metal-organic frameworks for targeted applications. *Acc. Chem. Res.* **50**, 805-813 (2017).

6. Tan, K. et al. Defect termination in the UiO-66 family of metal-organic frameworks: The role of water and modulator. *J. Am. Chem. Soc.* **143**, 6328-6332 (2021).

7. Shearer, G. C. et al. Defect engineering: tuning the porosity and composition of the metal-organic framework UiO-66 via modulated synthesis. *Chem. Mater.* **2016**, 28, 3749-3761.

8. Xu, C. Y. et al. Turning on visible-light photocatalytic C-H oxidation over metal-organic frameworks byintroducing metal-to-cluster charge transfer. *J. Am. Chem. Soc.* **141**, 19110-19117 (2019).

9. Zhao, W. S. et al. Fe-O clusters anchored on nodes of metal-organic frameworks for direct methane oxidation. *Angew. Chem. Int. Ed.* **60**, 5811-5815 (2021).

10. Ma, X. et al. Modulating coordination environment of single-atom catalysts and their proximity to photosensitive units for boosting MOF photocatalysis. *J. Am. Chem. Soc.* **143**, 12220-12229 (2021).

11. Abdel-Mageed, A. M. et al. Highly Active and stable single-atom Cu catalysts supported by a metal-organic framework. *J. Am. Chem. Soc.* **141**, 5201-5210 (2019).

12. Ye, G. et al. In situ implanting of single tungsten sites into defective UiO-66(Zr) by solvent-free route for efficient oxidative desulfurization at room temperature. *Angew. Chem. Int. Ed.* **60**, 20318-20324 (2021).

13. Kudin, K. N. et al. Raman spectra of graphite oxide and functionalized graphene sheets. *Nano Lett.* **8**, 36-41 (2008).

14. Zhang, T. et al. Overcoming acidic H_2O_2/Fe(II/III) redox-induced low H_2O_2 utilization efficiency by carbon quantum dots Fenton-like catalysis. *Environ. Sci. Technol.* **56**, 2617-2625 (2022).

15. Tian, X. et al. Hydroxyl radical-involving p-Nitrophenol oxidation during its reduction by nanoscale sulfidated zerovalent iron under anaerobic conditions. *Environ. Sci. Technol.* **55**, 2403-2410 (2021).

16. Li, H., Shan, C. & Pan, B. Fe(III)-doped g-C_3N_4 mediated peroxymonosulfate activation for selective degradation of phenolic compounds via high-valent iron-oxo species. *Environ. Sci. Technol.* **52**, 2197-2205 (2018).

17. Zazo, J. A. et al. Chemical pathway and kinetics of phenol oxidation by Fenton's reagent. *Environ. Sci. Technol.* **39**, 9295-9302 (2005).

18. Boukhatem, H. et al. Photocatalytic activity of mont-La (6%)-$Cu_{0.6}Cd_{0.4}S$ catalyst for phenol degradation under near UV visible light irradiation. *Appl. Catal. B: Environ.* **211**, 114-125 (2017).

19. Chen, R. Z. & Pignatello, J. J. Role of quinone intermediates as electron shuttles in Fenton and photoassisted Fenton oxidations of aromatic compounds. *Environ. Sci. Technol.* **31**, 2399-2406 (1997).

20. Duesterberg, C. K. & Waite, T. D. Kinetic modeling of the oxidation of p-hydroxybenzoic acid by Fenton's reagent: implications of the role of quinones in the redox cycling of iron. *Environ. Sci. Technol.* **41**, 4103-4110 (2007).

21. Ma, J. et al. Fenton degradation of organic pollutants in the presence of low-molecular-weight organic acids: Cooperative effect of quinone and visible light. *Environ. Sci. Technol.* **40**, 618-624 (2006).

22. Zagorsky, A. L. & Toropov, D. K. Polyphenylene producing method. RU: EP1 857 481 A, 2007/47.

23. Zhang, A. J. et al. Oxidative polymerization of hydroquinone using deoxycholic acid supramolecular template. *Sci. China: Chem.* **55**, 830-835 (2012).

24. Gözmen, B. et al. Indirect electrochemical treatment of bisphenol A in water via electrochemically generated Fenton's reagent. *Environ. Sci. Technol.* **37**, 3716-3723 (2003).

25. Liu, R. L., Xu, Y. M. & Chen, B. L. Self-assembled nano-FeO(OH)/reduced graphene oxide aerogel as a reusable catalyst for photo-Fenton degradation of phenolic organics. *Environ. Sci. Technol.* **52**, 7043-7053 (2018).

26. Tang, J. & Wang, J. Metal organic framework with coordinatively unsaturated sites as efficient Fenton-like catalyst for enhanced degradation of sulfamethazine. *Environ. Sci. Technol.* **52**, 5367-5377 (2018).
27. Zhang, Y. et al. Weakly hydrophobic nanoconfinement by graphene aerogels greatly enhances the reactivity and ambient stability of reactivity of MIL-101-Fe in Fenton-like reaction. *Nano Res.* **14**, 2383-2389 (2021).
28. Wang, J. L. & Wang, S. Z. Effect of inorganic anions on the performance of advanced oxidation processes for degradation of organic contaminants. *Chem. Eng. J.* **411**, 128392 (2021).
29. Liang, H. et al. Bimetal-organic frameworks with coordinatively unsaturated metal sites for highly efficient Fenton-like catalysis. *Chem. Eng. J.* **414**, 128669 (2021).
30. Yang, X. et al. Multiple roles of dissolved organic matter in advanced oxidation processes. *Environ. Sci. Technol.* **56**, 11111-11131 (2022).
31. Zazo, J. A. et al. Evolution of ecotoxicity upon Fenton's oxidation of phenol in water. *Environ. Sci. Technol.* **41**, 7164-7170 (2007).
32. Xia, M. et al. A highly active bimetallic oxides catalyst supported on Al-containing MCM-41 for Fenton oxidation of phenol solution. *Appl. Catal. B: Environ.* **110**, 118-125 (2011).
33. Wei, X., Wu, H. & Sun, F. Magnetite/Fe-Al-montmorillonite as a Fenton catalyst with efficient degradation of phenol. *J. Colloid Interface Sci.* **504**, 611-619 (2017).
34. Leal, T. W. et al. Low-cost iron-doped catalyst for phenol degradation by heterogeneous Fenton. *J. Hazard. Mater.* **359**, 96-103 (2018).
35. Wang, J. et al. Interlayer structure manipulation of iron oxychloride by potassium cation intercalation to steer H_2O_2 activation pathway. *J. Am. Chem. Soc.* **144**, 4294-4299 (2022).
36. Ling, C. et al. Atomic-layered Cu_5 nanoclusters on FeS_2 with dual catalytic sites for efficient and selective H_2O_2 activation. *Angew. Chem. Int. Ed.* **61**, e202200670 (2022).
37. Yang, Z. et al. Singlet oxygen mediated iron-based Fenton-like catalysis under nanoconfinement. *Proc. Natl. Acad. Sci. USA.* **116**, 6659-6664 (2019).
